# Sevoflurane versus PRopofol combined with Remifentanil anesthesia Impact on postoperative Neurologic function in supratentorial Gliomas (SPRING): protocol for a randomized controlled trial

**DOI:** 10.1186/s12871-020-01035-5

**Published:** 2020-05-19

**Authors:** Yan Xing, Nan Lin, Ruquan Han, John F. Bebawy, Yuming Peng, Jiaxin Li, Xiaoyuan Liu, Yan Li, Jia Dong, Min Zeng, Manyu Zhang, Lanyi Nie

**Affiliations:** 1 Department of Anesthesiology, Beijing Tiantan Hospital, Capital Medical University, No.119, West Road 4th ring South, Fengtai District, Beijing, 100070 China; 2 Departments of Anesthesiology & Neurological Surgery, Northwestern University Feinberg School of Medicine, 251 E. Huron St., F5-704, Chicago, IL 60611 USA

**Keywords:** (3–10): propofol, Sevoflurane, General anesthesia, Total intravenous anesthesia, Supratentorial glioma, Craniotomy, Neurologic function

## Abstract

**Background:**

Patients with intracranial tumors are more sensitive to anesthetics than the general population and are therefore more susceptible to postoperative neurologic and neurocognitive dysfunction. Sevoflurane or propofol combined with remifentanil are widely used general anesthetic regimens for craniotomy, with neither regimen shown to be superior to the other in terms of neuroprotective efficacy and anesthesia quality. There is no evidence regarding the variable effects on postoperative neurologic and neurocognitive functional outcome under these two general anesthetic regimens. This trial will compare inhalational sevoflurane or intravenous propofol combined with remifentanil anesthesia in patients with supratentorial gliomas and test the hypothesis that postoperative neurologic function is equally affected between the two regimens.

**Methods:**

This is a prospective, single-center, randomized parallel arm equivalent clinical trial, which is approved by China Ethics Committee of Registering Clinical Trials (ChiECRCT-20,160,051). Patients with supratentorial gliomas diagnosed by magnetic resonance imaging will be eligible for the trial. Written informed consent will be obtained before randomly assigning each subject to either the sevoflurane-remifentanil or propofol-remifentanil group for anesthesia maintenance to achieve an equal-desired depth of anesthesia. Intraoperative intervention and monitoring will follow a standard anesthetic management protocol. All of the physiological parameters and other medications administered during the intervention will be recorded. The primary outcome will be neurologic function change assessed by National Institute of Health Stroke Scale (NIHSS) within 4 h after general anesthesia when observer’s assessment of alertness/sedation (OAA/S) reaches 4. Secondary outcomes will include NIHSS and modified NIHSS change 1 and 2 days after general anesthesia, hemodynamic stability, intraoperative brain relaxation, quality of anesthesia emergence, quality of anesthesia recovery, postoperative cognitive function, postoperative pain, postoperative neurologic complications, as well as perioperative medical expense.

**Discussion:**

This randomized equivalency trial will primarily compare the impacts of sevoflurane-remifentanil and propofol-remifentanil anesthesia on short-term postoperative neurologic function in patients with supratentorial gliomas undergoing craniotomy. The exclusion criteria are strict to ensure that the groups are comparable in all aspects. Repeated and routine neurologic evaluations after operation are always important to evaluate neurosurgical patients’ recovery and any newly presenting complications. The results of this trial would help specifically to interpret anesthetic residual effects on postoperative outcomes, and perhaps would help the anesthesiologist to select the optimal anesthetic regimen to minimize its impact on neurologic function in this specific patient population.

**Trial registration:**

The study was registered and approved by the Chinese Clinical Trial Registry (Chinese Clinical Trial Registry, ChiCTR-IOR-16009177). Principle investigator: Nan Lin (email address: linnan127@gmail.com) and Ruquan Han (email address: hanrq666@aliyun.com) Date of Registration: September 8th, 2016. Country of recruitment: China.

## Background

According to the statistical report released from Primary Brain and Central Nervous System Tumors Diagnosed in the United States in 2015, thirty out of every 100,000 people in the general population suffered from a primary intracranial tumor requiring craniotomy for tumor resection. Glioma accounted for 60.8% of all primary intracranial tumors [1], of which most of these operations require general anesthesia. These patients have vulnerable central nervous systems because of their intracranial tumors (e.g., gliomas are aggressive lesions that invade central neuronal structures anatomically, and at the neuropathologocial level disturb neuronal connections to impair neural function). Compared to intact brains, neuronal network connection disruptions from intracranial lesions and central nervous system inhibition by anesthetics yield a “double insult” to neurosurgical patients, so to speak, and may make them more sensitive to general anesthesia and surgery, leading to both short and long-term neurologic and neurocognitive dysfunction. Anesthesiologists attempt to choose the anesthetic regimen cautiously, taking into account anesthetics’ effects on cerebral physiology (e.g., intracranial pressure and cerebral perfusion pressure), as well as to minimize the seeming disturbance to neuronal function by facilitating rapid emergence and orientation during recovery. The less postoperative neuronal function is affected by anesthetics, the more accurately the patient’s intracranial disease-related status is reflected, and this is beneficial to neurosurgeons so as to correctly evaluate neural function. The optimal postoperative treatment based on neurological evaluation contributes to reduction of neurologic complications and is critical for long-term quality of life. Therefore, it is important to investigate how different mechanisms of general anesthesia affect postoperative neural function and anesthesia recovery in patients with supratentorial (frontal-parietal-temporal) gliomas in both a qualitative and quantitative (over time) manner, as this may guide anesthetic choice in supratentorial craniotomies for gliomas to ensure a reasonable, safe, and economical perioperative anesthetic regimen.

Inhalational anesthesia and intravenous anesthesia are widely used in current neurosurgical anesthesiology [2], and sevoflurane combined with remifentanil or propofol combined with remifentanil are both accepted as general anesthesia strategies. Comparative studies have been done to investigate the effects of different anesthetics on craniotomies [3], both primary and secondary outcomes focused on the changes in intraoperative physiological parameters [3, 4], laboratory results [3], and anesthesia recovery indices during emergence [5, 6]; however, neuronal disease-specific study is lacking, and there is no evidence related to how postoperative neurological function and neurocognitive outcomes are affected by different general anesthesia strategies [7]. Patients with neurological diseases, e.g., brain tumors, are usually excluded in studies examining the correlation between anesthesia and neural function [8], as those diseases are major confounders leading to nervous system dysfunction, however, anesthetic effects on neural function cannot be ignored in neurosurgical patients, let alone the previously mentioned “vulnerable” brain tumor patient who is even more susceptible to anesthetics [9]. Recent findings have reported patients with supplementary motor area (SMA) lesions presenting with intraoperative neurological deficits for awake craniotomy which could not be explained anatomically by their intact corticospinal tracts as detected by cortical stimulation or cortical mapping [10, 11], and these deficits were reversible after operations over time without further intervention. Although this finding may have been related to lesion location, which is called “SMA symptom”, this phenomenon cannot exclude the possibility of residual anesthetic-induced neurological deficits in both the intraoperative and early postoperative periods.

It has been reported that in some supratentorial brain mass patients, a small plasma concentration of sedative could significantly worsen neurologic deficits before any operative intervention [9], but there is no evidence related to how residual anesthetics affect neurologic deficits after operations. Repeated neurological evaluations after neurosurgeries can help to assess whether surgical interventions are successful and whether long-term neurologic outcomes are primarily related to diseases and surgeries, while short-term neurologic outcomes are subject to perioperative care beyond the above factors; despite only a few hours of surgical intervention for brain tumor, the overlap of general anesthesia and its effects, after surgery is completed, can linger for hours to days, which may worsen neurologic function and confuse providers. However, in the early postoperative stage, it is unknown whether sevoflurane combined with remifentanil and/or propofol combined with remifentanil could result in unexpected neurologic function deterioration, and whether one strategy is inferior or superior to another.

In this study, we will use sevoflurane combined with remifentanil or propofol combined with remifentanil to maintain anesthesia during craniotomy in patients with supratentorial gliomas to determine whether they have comparable effects on neurologic function and neurocognition in the early postoperative period. Since both sevoflurane and propofol are GABAergic anesthetics, we hypothesize that these two general anesthesia methods equivalently affect early postoperative neural function in this group of patients.

## Methods

### Study purpose

(1) Primary purpose: To investigate neurologic function in the early postoperative period for patients with supratentorial intracranial gliomas under inhalational general anesthesia compared to intravenous general anesthesia. The hypothesis is that the difference in postoperative National Institute of Health Stroke Scale (NIHSS or modified NIHSS) score changes under the two general anesthesia methods are not different, with the score difference within − 1 to 1.

(2) Secondary purpose: To compare the effects of inhalational general anesthesia versus intravenous general anesthesia on neurocognition, hemodynamics, cerebral physiology, anesthesia recovery quality, pain scores, anesthesia expenses, and stress responses in patients with supratentorial intracranial gliomas undergoing elective craniotomy.

### Trial design

The study is a prospective, single-center, open label, randomized parallel arm equivalent clinical trial comparing sevoflurane and propofol, both combined with remifentanil, general anesthesia (Sevoflurane-remifentanil versus Propofol-remifentanil group) in patients with supratentorial gliomas; it will be carried out in the neurosurgical operation room in Beijing Tiantan Hospital (a large city), Capital Medical University, Beijing, China.

### Populations

Adult male and female patients scheduled for elective craniotomy under general anesthesia with supratentorial (frontal-parietal-temporal) gliomas diagnosed by magnetic resonance imaging (MRI) are eligible for the study. All patients must sign institutional approval and informed consent before enrollment, the written informed consent will be obtained 1 day or a few days before operation, when patients are seen by anesthesiologists in the ward. The information and reasons why eligible patients are not recruited to the trial will be documented. The inclusion and exclusion criteria are listed in Table 1.

**Table 1:** Inclusion and exclusion criteria

Inclusion Criteria	
Patients aged between 18 to 65 years old with American Society of Anesthesiology (ASA) status I ~ II who are scheduled for elective craniotomy for the treatment of supratentorial gliomas must fulfill the following:	
1	Frontal-Parietal-Temporal glioma diagnosed by preoperative MRI
2	Glasgow score of 15 without preoperative symptomatic elevated intracranial pressure
3	New and/or recurrent intracranial gliomas are allowed
Exclusion Criteria	
1	Unable to comprehend and cooperate with the neurologic examination
2	Emergency craniotomy or changed to emergency from elective craniotomy
3	Insular lobe is invaded by glioma
4	Scheduled intraoperative motor evoked potential monitoring
5	Patients with traumatic brain injury, intracerebral hemorrhage, or cerebral vascular diseases
6	Patients with prolonged emergence, postoperative mechanical ventilation, and/or sedation dependence due to a definite reason (e.g., surgery itself or tumor location)
7	Hypothalamic dysfunction
8	Radiotherapy and/or chemotherapy before surgery
9	Uncontrolled hypertension or severe heart disease that impairs cardiac function (New York Heart Association Functional Classification ≥ III)
10	History of related anesthetic allergy
11	Severe endocrine system dysfunction that impair metabolic index
12	Impaired mental status
13	Drug and/or alcohol abuse
14	Pregnant and/or lactation period patients
15	Neuromuscular diseases
16	Infectious and/or immune diseases with positive biomarker(s)
17	Body mass index > = 35

Patients will be recruited in Beijing Tiantan Hospital, Beijing, China. The potentially eligible patient will be screened and contacted by a trial team member who explains the study and ascertains the patient’s interest 1 or 2 days before the scheduled operation day. If interested in enrolment, the patient will receive the detailed trial explanation and written consent form.

### Randomization and blinding

Permuted-block randomization will be used with a block size of 4 and an allocation ratio of 1:1 to either sevoflurane-remifentanil group or propofol-remifentanil group. Random allocation sequence will be based on a computer-generated random digits table. One investigator who will not participate in anesthetic management or follow-up will implement the randomization and enroll patients, and allocation will be concealed in a sealed opaque envelope until patient enters the operating room. Randomization will occur for patients conforming to the above criteria, and written informed consent will then be obtained from themselves or their next-of-kin. Since the two general anesthetic administration routes are distinct (inhalational versus intravenous), patients and anesthesiologists cannot be blinded. An independent team who is not involved in the intraoperative management will be in charge of postoperative follow-up and is blinded to the intervention.

### Interventions

#### Standard anesthetic management

On the day of operation, patients will be admitted into the operating room to be randomly assigned into either the sevoflurane-remifentanil or propofol-remifentanil group. Vital signs including electrocardiography (ECG), blood pressure (BP), heart rate (HR), pulse oxygen saturation (SpO2), end-tidal carbon dioxide (ETCO_2_), body temperature, and urine output will be monitored throughout the study. 100% fraction of oxygen will be provided by mask to the patients for preoxygenation for 5 min prior to anesthetic induction. No premedication will be given.

All patients will be induced with 0.3 μg/kg sufentanil, 2–2.5 mg/kg propofol, and 0.7–0.8 mg/kg rocuronium or cisatracurium. After tracheal intubation, mechanical ventilation will be established, at a tidal volume of 8-10 ml/kg, respiratory rate of 12–15/min, inspiratory:expiratory ratio of 1:2, fraction of inhaled fresh oxygen as 60%, and flow rate of fresh gas as 1.5 L/min. After induction, anesthesia will be maintained according to one of the two group allocations: (1) 6-8 mg/kg propofol with 0.05–0.2 μg/kg remifentanil for the propofol group or (2) 1.3–1.5 Minimum Alveolar Concentration (MAC) sevoflurane with 0.05–0.2 μg/kg remifentanil for the sevoflurane group. The dosage of anesthetic will be adjusted according to the bispectral index (BIS) value, which will be maintained between 40 and 60. 0.5% ropivacaine 1 to 2 mL for each injection point will be used for scalp nerves block before the start of surgeries. Sufentanil 5–10 mcg can be given, by anesthesiologist discretion, to alleviate potent stress responses when head pins are placed or scalp incision is performed, based on the hemodynamic parameters. Additional sufentanil bolus at the dose of 5-10mcg during operation is allowed, the last bolus should be given ahead of at least 60 min before expected end of surgery. The muscle relaxant cisatracurium will be infused at 0.1 mg/kg/h during the operation for all patients and stopped once the bone flap is secured. Propofol and sevoflurane will be reduced according to BIS and hemodynamic parameters once the bone flap is fixed and stopped at skin dressing.

At the end of the operation, ondansetron 4 mg will be prophylactically administered to all patients to prevent nausea and vomiting, and tramadol 1.5-2 mg/kg will be given if patients are experiencing rigors/chills. Neostigmine 20-40mcg/kg and atropine 0.5-1 mg will be available to antagonize residual muscle relaxation when deemed necessary by train-of-four (TOF) twitch monitoring. Peripheral nerve stimulator will be performed for TOF monitoring, achieving TOF ratio of 1.0 at the ulnar nerve/adductor pollicis is considered an adequate sign of recovery from muscle relaxant.

Adverse hemodynamic responses will be recorded and classified as hypertension, hypotension, tachycardia, or bradycardia, which require standard treatment according to the protocol (Table 2). These episodes of blood pressure and heart rate changes and the medication administered during their treatment will be recorded as “hypertension” (MAP > 20% above preoperative baseline value) or “hypotension” (MAP < 20% below preoperative baseline value), Tachycardia and bradycardia are defined as HR > 100 bpm and HR < 45 bpm, respectively. Standard treatment for hemodynamic disturbances are shown in Table 2.

**Table 2:** Treatment for hemodynamic disturbances

Hemodynamic Fluctuation	Definition (if any of the below changes are sustained for equal and/or longer than 5 min)	Standard Treatment Algorithm
Hypertension Episode	MAP > 20% above preoperative baseline	Increase propofol or sevoflurane concentration according to BIS, increase remifentanil infusion rate, or administer 5mcg sufentanil; if correction still not achieved, nicardipine will be given as bolus and/or infusion
Hypotension Episode	MAP < 20% below preoperative baseline	Decrease propofol or sevoflurane concentration according to BIS, give adequate volume loading; if correction still not achieved, vasoactive agent administration (dopamine, norepinephrine, or phenylephrine) will be given with the dose and infusion rate adjusted according to the blood pressure response
Tachycardia Episode	HR > 100 bpm	Esmolol bolus and/or infusion according to heart rate response
Bradycardia Episode	HR < 45 bpm	Atropine administration

*MAP* mean arterial pressure, *HR* heart rate, *bpm* beat per minute

Patient-controlled intravenous analgesia (PCIA) will be used for postoperative pain control with 100mcg sufentanil and 16 mg ondansetron diluted to a total volume of 100 ml within normal saline being prepared, with a bolus dose set at 0.5 ml, a lockout time set at 15 min, and a background infusion of 2 ml/h. PCIA will be started after the patient is discharged from the operating room.

#### Anesthesia monitoring

Anesthesia management will aim to achieve targeted physiological parameters. The blood pressure will be monitored by radial artery catheter placement, and the target value is defined as the range of ±20% of the baseline MAP value, which is defined as the average MAP of the first three values measured after the patient enters the operating room and before induction. When the blood pressure is out of this range, measures will be taken such as changing the infusion rate of crystalloid or colloid and remifentanil, or giving bolus injection of sufentanil or vasoactive agent (such as 0.5 μg/kg/min phenylephrine or 0.01 μg/kg/min norepinephrine). Heart rate will be maintained between 50 and 90 beats per minute. SpO2 will be kept ≥98% during the operation. End-tidal CO_2_ will be maintained between 30 and 35 mmHg by adjusting ventilation parameters. Core body temperature will be kept between 36 and 37 degree Celsius. Plasma glucose concentration will be maintained between 5.0–7.8 mmol/L.

### Discontinuation criteria

#### Massive hemorrhage or massive transfusion

Massive intraoperative bleeding can occur, and this study defines massive bleeding as blood loss exceeding the entire blood volume within 24 h from the start of operation or 50% of circulating blood volume loss within 3 h during operation requiring emergency intervention. Intraoperative massive transfusion (transfusion of more than 10 units of packed red blood cells) may be needed if massive hemorrhage is encountered. If bleeding occurs to this extent and meets either or both of the above criteria, the trial will be discontinued for this particular patient.

#### Anaphylaxis during the operation

Anaphylaxis rarely but occasionally occurs intraoperatively, especially in the setting of antibiotic and muscle relaxant administration, but also with the administration of other medications, and normally can be treated quickly. If such a reaction occurs and is severe, necessitating discontinuation of the operation, the patient will be withdrawn from the trial. Anaphylaxis will be reported as an adverse event.

#### Venous air embolism

Significant venous air embolism has been observed in rare cases, but neurosurgery carries the potential risk for this sometimes intractable complication, especially when the operative intervention occurs near venous sinuses or in the sitting position [12–14]. Venous air embolism may result in severe hypoxia and hypotension which can impair cerebral perfusion and oxygenation. If this happens, timely intervention and resuscitation needs to be carried out and the patient will be withdrawn from the trial. This will be reported as an adverse event.

#### Postoperative coma

If the patient is in a coma (nonresponsive) after operation for any reason, the subject will not be able to cooperate with any neurological assessment, the subsequent follow-up will need to be discontinued, and this will be noted in the neurologic complication section. The patient will be withdrawn from the trial.

#### Outcomes

**Fig. 1:**
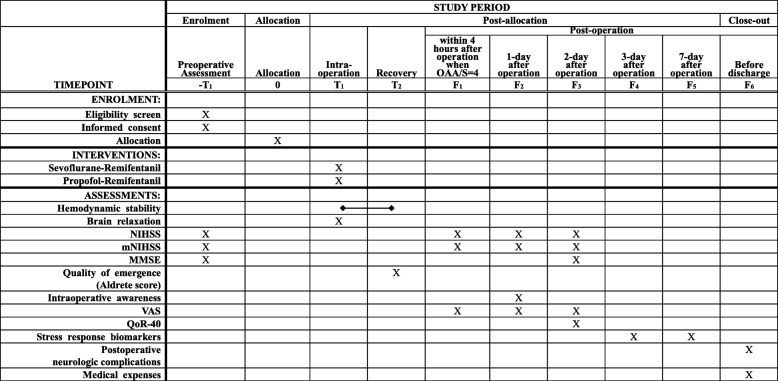
Schedule of enrollment, interventions and assessments for the SPRING trial

Patients will have been screened 1 to 2 days before operation, and the demographic characteristics, clinical manifestations, and past history obtained at this time. Detailed descriptions of the lesion in MRI will be obtained. The lesions’ pathologic diagnoses will be obtained 2 weeks after the tumor removal. Neurologic function will be assessed using the National Institute of Health Stroke Scale (NIHSS/mNIHSS) at baseline and in the postoperative period. NIHSS scores 15 items including consciousness, visual function, facial and motor function, ataxia, sensory and language function, and attention, and the total score ranges from 0 (no deficit) to a maximum of 42. A modified version of the NIHSS (mNIHSS) abridges to 11 items by deleting level of consciousness, facial palsy, limb ataxia, and dysarthria; the sensory item is collapsed from 3 to 2 choices [15]. Since there is no reliability and validity evidence comparing NIHSS and mNIHSS use in the intracranial tumor population, we will score both as primary outcomes in this study. The scoring system is shown in appendix. Mini-mental state examination (MMSE) will be used to assess the cognitive performance of patients at baseline and after operation. During anesthesia recovery, Anesthesia Recovery Quality and the modified Aldrete scale will be used to assess the recovery condition of patients. Visual Analog Scale (VAS) will be used to assess postoperative pain. QoR-40 (Quality of Recovery from Anesthesia) will be used at 2 days after operation to assess the recovery condition and the satisfaction of the patient related to anesthesia. All adverse events will be recorded. The schedule of data collection is shown in Fig. 1.

### Study endpoint

The primary endpoint will be NIHSS score change within 4 h after general anesthesia by sevoflurane-remifentanil or propofol-remifentanil when observer’s assessment of alertness/sedation (OAA/S) reaches 4. The time to reach OAA/S score of 4 at the first time point will be recorded.

Secondary endpoints will be as follows:

1. The NIHSS score change at postoperative day 1 and postoperative day 2 from the baseline, the level of OAA/S in these two time points will be recorded.
2. Hemodynamic stability including blood pressure, heart rate, episodes of hypertension, hypotension, tachycardia, and bradycardia;
3. Intraoperative brain relaxation (Brain relaxation assessed immediately after opening of the dura on a scale ranging from 1 to 4 (1 = perfectly relaxed, 2 = satisfactorily relaxed, 3 = firm brain, 4 = bulging brain);
4. Quality of anesthesia emergence including time of eye opening, time of emergence, coughing during extubation, postoperative nausea and vomiting (PONV), shivering, and agitation;
5. Quality of anesthesia recovery assessed by QoR-40 at postoperative day 2;
6. Changes of cognitive function at postoperative day 2 assessed by MMSE;
7. Postoperative pain at PACU, 1, and 2 days after surgery evaluated by VAS;
8. Postoperative neurological complications (e.g., intracranial hematoma, infection, seizure etc.);
9. Anesthesia expenses and in-hospital total expenses;

### Data collection and management

Anesthesiologists on the follow-up team are all trained and received certification from the NIHSS official training program website (www.nihstrokescale.org). Data collection ends when patients are discharged from the hospital. All the information will be recorded on a Case Report Form (CRF), and raw, non-numerical data are coded for data storage, review, tabulation and analysis. Data will be entered at our medical center and stored and monitored securely in an electronic databases. Each of the data collection forms and the detailed information will be discussed on an item-by-item basis. Double data entry will be used, different data entering individuals will use standardized terminology and abbreviations, training will be performed regarding entering data on forms, and we will respond promptly to data discrepancy queries and general concerns about overall quality. Any missing data or errors in the data will be summarized along with detailed descriptions, and will be queried by checking the original forms. Data safety and monitoring inspectors will evaluate the trial safety, efficacy, and any ethical issues. The data to be collected and the procedures to be conducted at each visit will be reviewed in detail. There will not be a formal data monitoring committee (DMC) in this study, because the intraoperative anesthesia management is standard with minimal risks and the follow-up duration is short for this trial.

Paper case report forms are stored in numerical order and kept in locked cabinets. The electronic data will be saved in a database with password protection, and the passwords will be changed on a regular basis. Database backup will be performed once a month. All the original files will be maintained in storage for a period of 5 years after completion of the study.

### Participant retention

Once a patient is enrolled in the study, the team will make every effort to follow the patient for the entire study period. For patients whose consent is withdrawn, who are lost to follow-up, or discontinue for any reason, the reason will be recorded in the CRF in order to better interpret the results. The strategies that are used to improve participant retention are:

1. provide letters regarding the evaluation time schedule prior to data collection to remind patients and relatives of each assessment and test;
2. provide adequate communication when visiting patients in the ward to let them be informed of upcoming data collection;
3. limit patient burden related to follow-up visits to the greatest extent possible;
4. patients who withdraw for follow-up assessment for one of the primary or secondary outcomes can continue with assessments for the other outcomes.

### Sample size calculation

Sample size calculation was conducted by PASS software (NCSS, LLC, USA). The mean difference of NIHSS change between the two types of anesthetics was estimated as 0.5, the estimated within group standard deviation was 1.5, and 1.0 was set as the equivalent margin based on clinical significance. Z-test was used as power analysis of equivalence tests of two independent proportions to detect equivalence. The minimum number of cases was 160 per group to achieve 80% power to detect a possible equivalence between the two groups. Considering the possibility of early termination during the study and allowing for a 10% drop-out rate, this number was raised to 176 subjects for each group. As two groups were to be studied, we aim to enroll 352 subjects.

### Statistical analysis plan

Kolmogorov-Smirnov testing will be used for evaluating if the continuous variables have a normal distribution or not. Descriptive statistics will be reported as means with standard deviation for normally distributed data; medians with interquartile range will be described for non-normally distributed data; for categorical variables, count (percentage) will be described. The difference in postoperative NIHSS scores at certain time points between the propofol and sevoflurance groups will be analyzed by t-test or Mann-Whitney U test depending on normal or non-normal distribution, respectively, and 95% confident interval will be determined for the NIHSS score change and difference between the two groups. The changes in VAS scores, intraoperative parameters, and stress response biomarkers over the observation time will be compared using repeated measurement, and post hoc analysis will be used for sensitivity analysis. For other secondary outcomes such as brain relaxation, MMSE score, anesthesia quality score and medical expense, the difference between the two groups will be analyzed by t-test or Mann-Whitney U test. All the categorical variables will be analyzed by chi-square testing. Missing data will be adjusted using inverse probability weighting and worst-case imputation scenarios. Statistical significance is defined as a type I error of 0.05, and the nature of the testing is two-tailed. Analyses will be conducted using Statistical Package for the Social Sciences (SPSS) version 17.0 (Chicago, IL, USA).

All data will be analyzed according to an intent-to-treat, such that all randomized patients originally allocated to the propofol or sevoflurane anesthesia arm at the time of randomization will be used for efficacy analysis. No interim analysis is planned. Worst observation carried forward method will be used to handle missing data.

### Reporting of adverse events

All adverse events associated with this trial will be closely monitored until the adverse events are resolved, stable, or confirmed as having no relation to the trial. Once adverse events occur, they will be immediately reported to the research department and to the principal investigator to determine the severity of the adverse events and the consequences of the injury. All adverse events associated with this study will be recorded and reported to the Ethics Committee within 1 week, which will be part of the annual report. The principal investigator will be responsible for all reported adverse events.

### Protocol amendment

The principle investigator of the SPRING trial will be responsible for any decision to amend the protocol. If there is any modification that impacts the conduct of the study and affects potential safety or benefit to the patient, such as trial objectives, design, patient population, sample sizes or study procedures in the protocol, the principle investigator will notify and gain approval from the China Ethics Committee of Registering Clinical Trials prior to implementation, and the related trial registration information will be updated in the Chinese Clinical Trial Registry.

### Trial results and publication

Publications and presentations related to this trial will always maintain and protect the integrity of the major objectives of this study. Each paper or abstract will be reviewed and approved by the principle investigator before being submitted. The SPRING trial may terminate at a planned target of 6 months after the last patient has been randomized. We expect to take about 4 to 6 months to compile the final results for publication in an appropriate medical journal. The results will be reported and disseminated to the public regardless of the magnitude or direction of effect. The principle investigator of the study should be considered as the lead author. All professionals that have participated in the SPRING trial for a minimum of 3 months will be listed in the authorship, and those who do not fulfill such criteria will be acknowledged in the publication.

## Discussion

In current neurosurgical anesthesia practice for elective craniotomy for brain tumor resection, either an inhalational agent or intravenous propofol in combination with a short-acting opioid and muscle relaxant may be used. For patients with normal intracranial pressure and those without need of intraoperative neurophysiological monitoring, there does not appear to be one technique that is deemed superior to another in terms of neuroprotective efficacy, anesthesia maintenance efficacy, or postoperative recovery quality. With respect to neurofunctional outcome, so far, no clinical evidence exists to elucidate whether there is a difference or equivalence between these two general anesthetics. Since both sevoflurane and propofol are GABAergic general anesthetic that have similar mechanism, we hypothesize that these two general anesthesia methods equivalently affect early postoperative neural function in patients with supratentorial gliomas.

We chose sevoflurane in one arm of this study as it is one of the most frequently used inhalational agents in our medical center, and worldwide. Compared to isoflurane and desflurane, it has the least vasoactive (vasodilatory) effect and can preserve cerebral blood flow best up to 1–1.5 MAC, while maintaining cerebral autoregulation [16–19]. Propofol is the only available intravenous general anesthetic for maintenance in both TIVA and combined inhaled-intravenous anesthesia. It is pharmacologically difficult to use only a single anesthetic agent to maintain general anesthesia during surgery, as usually an intraoperative analgesic is necessary as well, and so we selected an ultra short-acting opioid (remifentanil), which has a very short elimination half-life, so as to avoid respiratory depression postoperatively, which is especially important for neurosurgical patients.

The 15-item National Institutes of Health Stroke Scale (NIHSS) was developed for and is widely used to evaluate neurologic function [20]. Although the modified NIHSS (mNIHSS) appears to be a more easily-used measure that is identical with the original NIHSS in its validation and reliability when evaluating the severity of stroke [21, 22], the deletion of limb ataxia in the mNIHSS has significance in patients with intracranial tumors based on our previous study [9], and there is no evidence showing the superiority of one to another in the intracranial tumor population. We therefore will choose to calculate both the NIHSS and the mNIHSS for comprehensive neurologic function evaluation. The Chinese version of NIHSS has been validated in a previous study [23].

Motor evoked potential (MEP) monitoring is very important for some supratentorial craniotomies if the tumor is located in or near the motor strip or descending motor pathways near the prefrontal cortex. Clinical evidence shows that sevoflurane suppresses MEP amplitudes in a dose-dependent manner [24, 25]. When using a sevoflurane-propofol combination in clinical practice, sevoflurane concentration is usually maintained below 0.4 MAC; while in this study, sevoflurane will be the only general anesthetic to maintain the desired anesthetic depth in the sevoflurane arm, the concentration will be much higher and vary among individual patients, resulting in failure of MEP monitoring. Therefore, patients who require intraoperative neurophysiological monitoring will be excluded from the study.

The current study aims primarily to elucidate the possible differential effect of inhalational sevoflurane versus intravenous propofol anesthesia on the short period of post-operative neurologic function in patients receiving supratentorial tumor resection in a randomized controlled trial. As the repeated neurologic function assessments are important to evaluate the patients’ recovery from surgical intervention and the presence of any complications, the results of this trial would help to interpret anesthetic residual effects on postoperative outcomes, and perhaps help to determine the intraoperative general anesthetic that affects neurologic function with the least detriment and therefore optimizes anesthetic management in this specific patient population.

### Trial status

This registered study (ChiCTR-IOR-16009177) started recruiting on May 28th, 2018, and is planned to complete recruiting on December 31st, 2020. The protocol version number is 01 (September 1, 2016).

## Data Availability

The datasets used and analyzed during the current study are available from the co-corresponding author (Nan Lin, linnan127@gmail.com) on reasonable request.

## Appendix

**Table 3:** NIHSS and mNIHSS score system

Item	NIHSS	mNIHSS	Score
1a	LOC	[deleted]	0 = Alert 1 = Arousable by minor stimulation 2 = Arousable by strong/repeated stimulation 3 = Unresponsive
1b	LOC questions		0 = Answers both correctly 1 = Answers one correctly 2 = Answers neither correctly
1c	LOC Commands		0 = Performs both tasks correctly 1 = Performs one task correctly 2 = Performs neither task
2	Gaze		0 = Normal 1 = Parial gaze pulsy 2 = Forced deviation
3	Visual		0 = Normal 1 = Partial hemianopia 2 = Complete hemianopia 3 = Bilateral hemianopia
4	Facial Palsy	[deleted]	0 = Normal 1 = Minor 2 = Parial 3 = Complete
5a	Motor Arm (left)		0 = No drift 1 = Drift before 10s 2 = Falls before 10s 3 = No effort against gravity 4 = No movement UN = Amputation or joint fusion explain:
5b	Motor Arm (right)		
6a	Motor Leg (left)		
6b	Motor Leg (right)		
7	Limb Ataxia	[deleted]	0 = Absent 1 = In one limb 2 = In two Limb UN = Amputation or joint fusion
8	Sensory		In NIHSS: 0 = Normal 1 = Mild-to-Moderate loss 2 = Severe to total loss In mNIHSS: 0 = Normal 1 = Abnormal
9	Language		0 = Normal 1 = Mild-to-Moderate aphasia 2 = Severe aphasia 3 = Mute or global aphasia
10	Dysarthria	[deleted]	0 = Normal 1 = Mild-to-Moderate 2 = Severe UN=Intubated
11	Extinction and Inattention		0 = Normal 1 = Visual, tactile, auditory, spatial, or personal inattention 2 = Profound hemi-inattention or extinction to more than one modality

*NIHSS* National Institute of Health stroke scale, *mNIHSS* modified National Institute of Health stroke scale, *LOC* Level of Consciousness

The total score in NIHSS and mNIHSS are 42 and 31 respectively

## Abbreviations

BP: Blood pressure
BIS: Bispectral index
ECG: Electrocardiography
ETCO_2_: End-tidal carbon dioxide
GABA: Gamma-aminobutyric acid
HR: Heart rate
LOC: Level of consciousness
MAC: Minimum alveolar concentration (the concentration of an inhalational anesthetic in the alveoli of the lungs that is needed to prevent movement in 50% of subjects in response to surgical stimulus.)
MAP: Mean arterial pressure
MEP: Motor evoked potential
MMSE: Mini-mental state examination
MRI: Magnetic resonance imaging
NIHSS: National Institute of Health stroke scale
mNIHSS: Modified National Institute of Health stroke scale
OAA/S: Observer’s assessment of alertness/sedation
PCIA: Patient-controlled intravenous analgesia
SMA: Supplementary motor area
SpO_2_: Pulse oxygen saturation
VAS: Visual analog scale
